# A comparative study of domestic decision-making power and social support as predictors of postpartum depressive and physical symptoms between immigrant and native-born women

**DOI:** 10.1371/journal.pone.0231340

**Published:** 2020-04-08

**Authors:** Hung-Hui Chen, Li-Yin Chien

**Affiliations:** 1 School of Nursing, College of Medicine, National Taiwan University, Taipei, Taiwan; 2 Department of Nursing, National Taiwan University Hospital, Taipei, Taiwan; 3 Institute of Community Health Care, School of Nursing, National Yang-Ming University, Taipei, Taiwan; University of Michigan, UNITED STATES

## Abstract

**Background:**

Women’s participation in decision-making in the household is an indicator of women’s empowerment. Few studies have compared domestic decision-making power and its effect on postpartum health between immigrant and native-born women. This study aimed to examine the effect of domestic decision-making power and social support during pregnancy on predicting postpartum depressive and physical symptoms among immigrant and native-born mothers in Taiwan.

**Methods and findings:**

This prospective study recruited 177 marriage-based immigrant mothers and 230 native-born women who were at least twelve weeks pregnant from hospitals, clinics and health centers. Data were collected in the 2nd or 3rd trimester of pregnancy and at 3 months postpartum from March 2013 to March 2015. Postpartum depression and the severity of postpartum physical symptoms were measured using the Edinburgh Postnatal Depression Scale, and a 17-item, 4-point Likert scale, respectively. Linear regression was used to examine the relationship between “domestic decision-making power and social support during pregnancy” and “depressive and physical symptoms at 3 months postpartum.” Women who had lower domestic decision-making power and social support during pregnancy had higher postpartum depressive and physical symptoms. Those women with full-time employment and insufficient family income had higher postpartum depressive symptoms. Though immigrant women scored lower in domestic decision-making power and social support than native-born women, they had lower mean scores in postpartum depressive and physical symptoms. After accounting for the abovementioned factors, immigrant women remained at lower risk for postpartum depressive and physical symptoms than native-born women. There was significant interaction between domestic decision-making power and immigrant status, suggesting that the association between domestic decision-making and postpartum depressive and physical symptoms was smaller for immigrants than for native women.

**Conclusions:**

Domestic decision-making power and social support during pregnancy are protective predictors of postpartum depressive and physical symptoms. However, the effect of domestic decision-making power appeared to be less salient for immigrants, probably due to the “healthy immigrant effect” and/or lower expectations toward domestic decision-making power among immigrants. The finding that immigrant women demonstrated a lower level of domestic decision-making power suggests that empowerment issues need to be addressed among immigrants.

## Introduction

The number of international migrants worldwide reached 258 million in 2017 [1], and it continues to increase. Marriage-based immigration has grown around the world [2,3]. Marriage migration has also become common in Southeast and East Asia. Many of the women who come from Southeast and East Asia are married to men in Singapore, South Korea, Japan, or Taiwan, and they migrate to their husbands’ country [4–6]. Of the immigrants who have migrated to Asian countries, roughly 60% were born in Asia [1]. Evidencing the increasing rate of transnational marriage, more than 500 thousand women from Southeast Asia had married Taiwanese men and migrated to Taiwan by July 2019 [7]. Immigrant women in Taiwan composed nearly one-ninth of all marriages in 2018 [8]. Marriage-based immigrant women in Taiwan mostly come from China (67%), Vietnam (21%), Indonesia (6%), the Philippines, Thailand, Cambodia, or other countries [6%; 7]. These immigrant women appear to be younger, have lower education levels, and marry into families with lower socioeconomic statuses than native-born women. Some of these marriages are arranged through dealers and lack a solid basis of affection [9,10]. Immigrant women usually marry into relatively traditional and patrilineal families that view continuance of family lines to be the most important task [11,12]. Many immigrant women become pregnant soon after arriving in Taiwan [10]. Immigrant women’s pregnancies accounted for nearly one-thirteenth of all birth in Taiwan in 2018 [13].

A woman’s participation in domestic decision-making is a critical indicator of familial and/or interpersonal empowerment and status in a household [14]. The status of wives in the family is, on average, lower than that of husbands owing to traditional patrilineal cultures. Previous cross-sectional studies have shown that social support and domestic decision-making power levels are negatively associated with postpartum depressive symptoms. One study showed that immigrant women have lower domestic decision-making power over personal or child/family matters and a higher prevalence of postpartum depressive symptoms than native-born women [9]. Another cross-sectional study reported that immigrant mothers had more postpartum physical symptoms than did native-born women [15].

Little is known about the causal relationships between domestic decision-making power and postpartum health among immigrant and native-born women since previous studies applied a cross-sectional design. The objectives of this prospective study were to compare domestic decision-making power, social support, and postpartum depressive and physical symptoms between immigrant and native-born women; and examine the predictive relationships of domestic decision-making power and social support during pregnancy on postpartum depressive and physical symptoms among immigrant and native-born women in Taiwan.

## Methods

This prospective study was performed from March 2013 to March 2015. We recruited immigrant and native-born women to complete structured questionnaires in the second or third trimester of their pregnancies and at 3 months postpartum. A short description (including study purpose, assurances of confidentiality, and the right to refuse to participate) and a consent form were provided to the immigrant and native-born women. Those who agreed to participate in the study signed a consent form and provided their contact information. The study participants were interviewed face-to-face or via telephone during their pregnancies. After delivery, they were permitted to choose the mode of data collection: face-to-face, by telephone, postal mail-in, or via email.

In Taiwan, the majority of immigrant women came from China (about two-third) and Vietnam (about one-fifth). For immigrants from China, no problem was found in communicating using Chinese language. We prepared the Vietnamese questionnaire for immigrants from Vietnam. The interviewer was accompanied by a trained interpreter when immigrant women were not able to communicate in Chinese. In the first interview, all immigrants’ Chinese language ability was assessed. For those immigrants whose Chinese language ability was limited, face-to-face or telephone interview accompanied by a translator was arranged for follow-up.

A gift voucher worth one hundred New Taiwan dollars was provided to study participants after completing the questionnaire each time. The study protocol was approved by the institutional review boards at Mackay Memorial Hospital, Tzu Chi General Hospital Taipei Branch, Taipei City Hospital, and Saint Mary’s Hospital Luodong.

### Study participants

The study population was adult women who were at least 20 years of age, in the second or third trimester of pregnancy, and currently living in Taiwan. The immigrant and native-born women were recruited from 2 obstetrical clinics, 4 hospitals, and 25 Health Centers across Taiwan. The term “immigrant women” refers to those women who were born outside of Taiwan and married to Taiwanese men. A total of 372 pregnant immigrant women and 466 pregnant native-born women were referred to the research team, of whom 62 immigrant women (16.7%) and 83 native-born women (17.8%) refused to participate in the study. A total of 310 immigrant and 383 native-born women completed the interview during pregnancy, of whom 177 immigrant (43.5%) and 230 native-born (56.5%) women completed the interview at 3 months postpartum. The final sample for the present analyses included 407 participants, all of whom completed interviews during pregnancy and at 3 months postpartum (177 immigrant and 230 native-born women). We compared those who completed the interviews during pregnancy and at 3 months postpartum with those who were lost to follow-up, and no significant differences were found in age, perceived family income sufficiency, parity, infant gender, marital satisfaction, history of depression diagnosis, or depressive symptomatology during pregnancy.

### Measurements

The study variables included socio-demographics (age, immigrant status, educational level, spouse’s educational level, work status during pregnancy, perceived family income sufficiency), obstetric variables (parity, infant gender), marital satisfaction, original nationality of immigrants, history of depression diagnosis, and depressive symptoms, decision-making power, and social support during pregnancy. Postpartum depressive and physical symptoms were measured at 3 months postpartum.

Perceived family income sufficiency was measured with a 5-point Likert scale ranging from very insufficient (0) to very sufficient (4), according to the participants’ perception of the sufficiency of their family income for daily expenses. Marital satisfaction was rated on a 5-point Likert scale ranging from 0 (very dissatisfied) to 4 (very satisfied).

The concept of social support included emotional, instrumental, and informational subcategories. Each dimension of support was measured using a 4-item, 5-point Likert scale. Therefore, total scores of social support ranged from 0 to 48, with higher scores indicating better social support. A previous study examined the validity and reliability of the social support scale [16]. The social support scale has been applied in previous studies of immigrant and native-born women [9,17]. The internal consistency of the scale in this study was assessed using Cronbach’s alpha and was 0.944 (0.957 for immigrant women and 0.927 for native-born women).

Domestic decision-making power during pregnancy was measured with a 9-item, 5-point Likert scale. Total scores ranged from 0 to 36, with higher scores indicating higher decision-making power. The scale was modified from the previous version, which was a 7-item scale [9], 2 items were added to address the circumstance of pregnancy. This scale measures women’s domestic decision-making power in personal matters (5 items) and child and family matters (4 items). Principal components factor analysis with varimax rotation, a loading criterion of 0.5, and Kaiser’s eigenvalue of 1 or greater were conducted on each item. The result of the factor analysis supported the same 2-factor structure as in the previous version. The items and factor loadings are displayed in Table 1. The internal consistency of the scale, using Cronbach’s alpha, was 0.922 (0.931 for immigrant women and 0.914 for native-born women).

**Table 1:** Factor analysis for the domestic decision-making power scale (N = 407).

	Factor loadings	
	Over personal matters	Over child and family matters
1. Have your own money and can spend it	**0.798**	0.357
2. Need consent to buy personal things	**0.824**	0.359
3. Need consent to buy household groceries	**0.867**	0.286
4. Need consent to buy baby products	**0.863**	0.228
5. What to eat and do	**0.775**	0.289
6. Number of births or children	0.337	**0.728**
7. Contraceptive methods	0.250	**0.770**
8. Obstetrician and prenatal exam	0.191	**0.585**
9. Need consent to go to work	0.253	**0.731**
Eigenvalue	5.157	1.005
% of variance	57.302	11.172

Depressive symptoms during pregnancy and postpartum were measured using the Edinburgh Postnatal Depression Scale [EPDS; 18,19]. EPDS includes 10 items, which are measured on a 4-point Likert scale, and results in a possible total score range from 0 to 30. EPDS was used to measure depressive symptoms over the 7 days prior to the interview. The validity and reliability of the EPDS were demonstrated among immigrant and native-born women in Taiwan [15, 18]. The internal consistency of the scale, using Cronbach’s alpha, was 0.916 (0.918 for immigrant women and 0.913 for native-born women) at postpartum. We used a cutoff score of 15 during pregnancy to indicate presence of depressive symptomatology during pregnancy [19]. Postpartum depressive symptoms were treated as a continuous variable with higher scores indicating more depressive symptoms [20,21].

The severity of postpartum physical symptoms was measured using a 17-item self-designed checklist. Each item score assigned to the Likert scale was 0 (none) to 3 (severe) for all but 2 questions (“varicose veins on legs” and “more colds than usual”). The items “varicose veins on legs” and “more colds than usual” were measured by dichotomized answers of no (0) and yes (3). The scores of all items were totaled to determine the cumulative severity of postpartum physical symptoms, with a possible range of 0 to 51 points. Higher scores indicate higher severity of postpartum physical symptoms. The scale has been applied in previous studies [22]. The internal consistency of the scale, using Cronbach’s alpha, was 0.785 (0.794 for immigrant women and 0.769 for native-born women).

### Data analysis

In this study, we used means and standard deviations (SD) to describe continuous variables and frequencies and percentages to describe categorical variables. Chi-squared tests or independent *t*-tests were used to compare independent and dependent variables between immigrant and native-born women. Multiple linear regression was used to examine the association between domestic decision-making power during pregnancy and “level of postpartum depressive and physical symptoms” after controlling for other significant variables. Stepwise methods in linear regression were applied to identify significant variables in the model. Statistical significance was determined by a two-tailed p-value of <0.05. We examined whether the estimates of the main variables in the final parsimonious model would change when age and history of depression diagnosis were adjusted in the model. Since there were little change in the estimates of the main variables, we decided to present the final parsimonious model in the paper. We performed the data analyses using IBM SPSS Statistics for Windows, Version 22.0 (IBM Corp., Armonk, NY, USA).

## Results

The socio-demographics, obstetric variables, and marital satisfaction of the immigrant and native-born women are compared in Table 2. Immigrant women (mean age = 30.03 years; SD = 4.562; range = 20.42–43.92 years) were significantly younger than native-born women (mean = 32.32 years; SD = 3.586; range = 22.25–43.08 years; *p*<0.001). More immigrant women and their spouses (55.1% and 36.9%) had an educational level of less than vocational school/university when compared with the native-born women and their spouses (10.9% and 16.3%; both *p*<0.001). Only 22% of immigrant women had full-time employment during pregnancy, which was significantly less than native-born women (about 75%; *p*<0.001). Perceived family income sufficiency and marital satisfaction did not differ to a statistically significant degree between immigrant and native-born women. About two-thirds of immigrant women came from China (125; 70.6%) and the rest came from other Asian countries (Vietnam: 22; Myanmar: 8; Indonesia: 9; Malaysia: 5; Philippines: 2; Cambodia: 2; Nepal: 1; Japan: 3).

**Table 2:** Characteristics of the study participants (N = 407).

	Immigrant women (*n* = 177)	Native women (*n* = 230)	*p*
Age (years)			<0.001***
20-<25	24 (13.6%)	5 (2.2%)	
25-<30	72 (40.7%)	46 (20.0%)	
30-<35	54 (30.5%)	127 (55.2%)	
≥35	27 (15.2%)	52 (22.6%)	
Educational level^a^			<0.001***
Elementary school or lower	6 (3.4%)	0 (0%)	
High school	91 (51.7%)	25 (10.9%)	
Vocational school/University	74 (42.1%)	159 (69.1%)	
Postgraduate	5 (2.8%)	46 (20.0%)	
Spouse’s educational level^a^			<0.001***
Elementary school or lower	3 (1.7%)	2 (0.9%)	
High school	62 (35.2%)	40 (17.4%)	
Vocational school/University	94 (53.4%)	132 (57.4%)	
Postgraduate	17 (9.7%)	56 (24.3%)	
Work status during pregnancy			<0.001***
None	123 (69.5%)	47 (20.4%)	
Part-time	15 (8.5%)	11 (4.8%)	
Full-time	39 (22.0%)	172 (74.8%)	
Perceived family income sufficiency			
Not enough	28 (15.8%)	41 (17.8%)	0.272
Just enough	80 (45.2%)	117 (50.9%)	
More than enough	69 (39.0%)	72 (31.3%)	
Parity			0.568
1	102 (57.6%)	139 (60.4%)	
>1	75 (42.4%)	91 (39.6%)	
Infant gender^b^			0.741
Female	82 (46.6%)	110 (48.2%)	
Male	94 (53.4%)	118 (51.8%)	
Marital satisfaction^c^			0.265
Dissatisfied	4 (2.3%)	5 (2.2%)	
Neither satisfied nor dissatisfied	40 (22.6%)	37 (16.2%)	
Satisfied	133 (75.1%)	186 (81.6%)	
History of depression diagnosis			0.193
No	175 (98.9%)	223 (97.0%)	
Yes	2 (1.1%)	7 (3.0%)	
Depressive symptomatology during pregnancy			0.013*
No	176 (99.4%)	219 (95.2%)	
Yes	1 (0.6%)	11 (4.8%)	

^a^N = 406

^b^N = 404

^c^N = 405

*p<0.05

***p<0.001

Immigrant women reported a significantly lower score in domestic decision-making power (28.72 vs. 30.73; *p* = 0.003) than native-born women. Though immigrant women also reported a lower score in social support, the difference did not reach statistical significance (31.56 vs. 33.13; *p* = 0.062). Immigrant women scored lower in all dimensions underlying domestic decision-making power and social support than did native-born women. However, only the differences in domestic decision-making power over child and family matters and instrumental support reached statistical significance (Table 3). The mean depressive symptoms score (6.19 vs. 7.71; *p* = 0.01) and the severity of physical symptoms score (5.15 vs. 7.26; *p*<0.001) at 3 months postpartum were significantly lower among immigrant women than they were among native-born women. The most prevalent physical symptom at 3 months postpartum was backache for both immigrant and native-born women. Native-born women reported higher severity of postpartum physical symptoms in backache, urinary incontinence, vaginal infections, excessive leucorrhea or vaginal discharges, numbness in the hands, numbness in the feet, cold hands and/or feet, and varicose veins on legs than did immigrant women (Table 3).

**Table 3:** Domestic decision-making power, social support, and postpartum depressive and physical symptoms (N = 407).

	Immigrant women (*n* = 177) mean (SD)	Native women (*n* = 230) mean (SD)	*p*
During pregnancy (2^nd^ or 3^rd^ trimester)			
Domestic decision-making power (total scale)	28.72 (7.630)	30.73 (5.522)	0.003**
Over personal matters	17.02 (4.722)	17.26 (3.255)	0.573
Over child and family matters	11.69 (3.794)	13.47 (2.677)	<0.001***
Social support (total scale)	31.56 (9.112)	33.13 (7.307)	0.062
Emotional support	11.24 (3.379)	11.50 (2.777)	0.402
Instrumental support	10.29 (3.910)	11.20 (3.232)	0.013*
Informational support	10.03 (3.812)	10.43 (2.952)	0.252
After delivery (at 3 months postpartum)			
Postpartum depressive symptoms	6.19 (6.091)	7.71 (5.732)	0.010*
Postpartum severity of physical symptoms	5.15 (4875)	7.26 (5.466)	<0.001***
Physical symptom	n (%)	n (%)	
Headache	44 (24.9%)	64 (27.8%)	0.501
Backache	113 (63.8%)	176 (76.5%)	0.005**
Dizziness	45 (25.4%)	73 (31.7%)	0.164
Hemorrhoids	57 (32.2%)	77 (33.5%)	0.789
Constipation	58 (32.8%)	96 (41.7%)	0.064
Urinary incontinence	12 (6.8%)	60 (26.1%)	<0.001***
Urinary tract infections	8 (4.5%)	11 (4.8%)	0.901
Vaginal infections	18 (10.2%)	51 (22.2%)	0.001**
Joint pain	53 (29.9%)	85 (37.0%)	0.138
Poor sleep quality or insomnia	107 (60.5%)	146 (63.5%)	0.533
Excessive leucorrhea or vaginal discharges	37 (20.9%)	80 (34.8%)	0.002**
Excessive vaginal bleeding	7 (4.0%)	16 (7.0%)	0.194
Numbness in the hands	35 (19.8%)	65 (28.3%)	0.049*
Numbness in the feet	19 (10.7%)	48 (20.9%)	0.006**
Cold hands and/or feet	27 (15.3%)	64 (27.8%)	0.003**
Varicose veins on legs	9 (5.1%)	49 (21.3%)	<0.001***
More colds than usual	25 (14.1%)	42 (18.3%)	0.265

*p<0.05

**p<0.01

***p<0.001

Multiple linear regression models for depressive symptoms at 3 months postpartum are presented in Table 4. The models showed that when considered together, immigrant and native-born women’s age, work status, perceived family income sufficiency, depressive symptomatology during pregnancy, social support, and domestic decision-making power were associated with level of postpartum depressive symptoms. Women who had full-time employment had higher postpartum depressive symptom scores than those who did not (β = 0.107; *p* = 0.044). Women who perceived that they had sufficient family income scored lower in postpartum depressive symptoms than those who perceived just enough or insufficient family income (β = -0.091; *p* = 0.047). Presence of depressive symptomatology during pregnancy (β = 0.246; *p*<0.001) and a lower level of social support (β = -0.233; *p*<0.001) were associated with an increased depressive symptom score at 3 months postpartum. Women who perceived a lower level of domestic decision-making power during pregnancy were more likely to have increased depressive symptoms (β = -0.184; *p* = 0.014). After accounting for these factors, immigrant women still had a lower level of postpartum depressive symptoms than did native-born women (β = -0.500; p = 0.023). We further analyzed whether immigrant status interacted with social support or domestic decision-making power in this multiple linear regression model. We found that the main effects of immigrant status and domestic decision-making power during pregnancy as well as their interaction terms were significant in the model. When the immigrant and native-born women were considered separately in the models, domestic decision-making power was significantly and negatively associated with postpartum depressive symptoms in the model of native-born women alone. Social support was significantly and negatively associated with postpartum depressive symptoms in the models for the immigrant and the native-born women, respectively.

**Table 4:** Linear regression model for depressive symptom scores at 3 months postpartum.

	Immigrant and native women		Immigrant women		Native women	
	β	*p*	Β	*p*	β	*p*
Immigrant women	-0.500	0.023*				
Work status: full-time	0.107	0.044*	0.140	0.061	0.057	0. 314
Perceived sufficient family income	-0.091	0.047*	-0.084	0.266	-0.099	0.084
Depressive symptomatology during pregnancy	0.246	<0.001***	0.063	0.391	0.328	<0.001***
Social support	-0.233	<0.001***	-0.209	0.006**	-0.264	<0.001***
Domestic decision-making power	-0.184	0.014*	-0.003	0.968	-0.140	0.019*
Immigrant women* Domestic decision-making power	0.547	0.042*				

CI = confidence interval.

*p<0.05

**p<0.01

***p<0.001

The multiple linear regression models for the severity of postpartum physical symptoms at 3 months postpartum are presented in Table 5. In the model for all women together, the native-born women (β = -0.751; *p* = 0.001), women who had depressive symptomatology during pregnancy (β = 0.106; *p* = 0.031), women who perceived a lower level of social support (β = -0.248; *p*<0.001) and women with a lower level of domestic decision-making power (β = -0.179; *p* = 0.020) were more likely to have more severe physical symptoms. We further analyzed the interaction effect. We found that immigrant status interacted with domestic decision-making power in this model. When the immigrant and native-born women were considered separately in the models, domestic decision-making power was significantly associated with postpartum physical symptoms among native-born women, but not among immigrant women. Social support was still a statistically significant predictor of postpartum physical symptoms for immigrant and native-born women.

**Table 5:** Linear regression model for severity of physical symptoms at 3 months postpartum.

	Immigrant and native women		Immigrant women		Native women	
	β	*p*	Β	*p*	β	*p*
Immigrant women	-0.751	0.001**				
Depressive symptomatology during pregnancy	0.106	0.031*	0.015	0.844	0.133	0.044
Social support	-0.248	<0.001***	-0.262	0.001**	-0.253	<0.001***
Domestic decision-making power	-0.179	0.020*	0.071	0.338	-0.136	0.038*
Immigrant women* Domestic decision-making power	0.547	0.015*				

CI = confidence interval.

*p<0.05

**p<0.01

***p<0.001

## Discussion

This study is unique in that it shows the temporal relationship between domestic decision-making power during pregnancy and postpartum depressive and physical symptoms. This prospective study demonstrated that domestic decision-making power during pregnancy was a protective factor for depressive and physical symptoms at 3 months postpartum. In addition, the interaction between immigrant status and domestic decision-making power during pregnancy was significant, meaning that the association between domestic decision-making power and postpartum depressive/physical symptoms differed between immigrant and native-born women. The association is less salient among immigrant women.

The less salient association between domestic decision-making power and postpartum depressive/physical symptoms could be due to the healthy immigrant effect [23,24]. Though some marriage-based immigrant women in Taiwan migrated for economic reasons, yet they have high levels of motivation and ambition to migrate based on our observation. Previous studies suggested that immigrants are on average healthier than the native population in the host country due to characteristics of mother country, immigrant culture as well as immigrant motivation and ambition [23, 24]. Our finding that immigrant women have fewer postpartum depressive and physical symptoms supports the assumption underlying the healthy immigrant effect, which posits that women who are able to migrate are generally healthy. Therefore, the effect on negative health outcomes is less apparent. Nevertheless, the lower prevalence of postpartum symptoms among immigrants differed from the findings of earlier studies in Taiwan, which showed that immigrant mothers had higher postpartum depressive symptoms and minor morbidities [9,15]. The differences could be attributed to several government policies aimed at helping immigrants in the most recent decade. They include fully-covered prenatal care and delivery services, a health center-based case management program, multilingual website and service line, translator services, and multi-cultural promotion activities. The policies may improve the perinatal health outcomes of immigrant women, and their improved health outcomes are making the healthy immigrant effect apparent. Moreover, these policies increase social support among immigrant women, and thus, the differences in overall social support between immigrant and native-born women are decreasing.

The other explanation for the lack of significant association between domestic decision-making power and postpartum depressive/physical symptoms among immigrants may be related to differences in expectation of domestic decision-making power between immigrant and native women. Perhaps immigrant women entered their marriages with little expectation of having power over domestic decisions, and therefore they did not feel distressed by its lack. This speculation is partly supported by the finding that marital satisfaction is similar between immigrant (satisfied: 81.6%) and native (75.1%) women, though immigrant women had significantly lower domestic decision-making power and social support than native women.

Social support and domestic decision-making power represent two important aspects of integration for newly arrived immigrants to their host society. Immigrant women showed significantly lower instrumental support than did native-born women. Together with the finding that immigrant women showed a significantly lower domestic decision-making power than native-born women, the results suggest that immigrant women assume more household work and have lower status in the household. Empowerment issues among the marriage-based immigration program may still need to be addressed, with emphasis on increasing their social support.

Depressive symptomatology during pregnancy, full-time employment, and perceive insufficient family income were related to a higher level of depressive symptoms at 3 months postpartum. The usual length of maternity leave after childbirth is about 8 weeks in Taiwan [25]. Working mothers may have a higher level of depressive symptoms in their first month of returning to work. Family income insufficiency causes great stress, which increases postpartum depressive symptoms [26]. Similar associations have been reported in previous studies [27, 28]. Policy support for working mothers and financial subsidies for families with young children may be implicated. Screening women with depressive symptoms during pregnancy and providing intervention accordingly could help improve their postpartum health.

In this study, we chose to use level of depressive symptoms and severity of physical symptoms as the dependent variables. In the scenario of women’s empowerment, we believe that postpartum well-being (continuous variable) rather than disease diagnosis (categorical variable based on the cut-off scores) is more relevant. Previous studies had used continuous scores to indicate level of postpartum symptoms [18, 19, 22]. To adjust for depressive symptoms during pregnancy, we dichotomized EPDS scores during pregnancy using a cut-off score of 15. This approach is to avoid the auto-correlation of depressive symptoms at different time points.

### Limitations

This study had several limitations. First, a relatively small sample size limited our ability to confirm the variables that had small associations with dependent variables as meaningful predictors when performing our statistical analyses. For example, we were not able to examine the interaction between social support and domestic decision-making power in relation to postpartum depressive/physical symptoms among immigrant women. Second, high attrition rate (41.3%) was shown in this study. The potential for selection bias was difficult to avoid because only those who completed all assessments were included in analyses. However, no differences were found between those who completed all assessments and those who did not (see Study participants/Methods). Third, the women’s depressive symptoms were measured by self-reported EPDS, which reflects the level of an individual’s depressive symptoms and is not a clinical diagnosis. Likewise, family income was self-reported as perceived family income sufficiency, not actual family income. This methodological aspect reflected the fact that many immigrant women were not aware of their actual family income. Fourth, the immigrant group was composed of women from different nationalities. Due to language barriers and response pattern, consistent measurement of postpartum depressive and physical symptoms among immigrant women of different cultural background may be a concern. Fifth, we did not exclude those who had a history of depression diagnosis and had depressive symptomatology during pregnancy due to limited sample size concern. However, we did adjust for depressive symptomatology during pregnancy in the model. Adding history of depression diagnosis or not to the final model did not change our results. Finally, potential confounders that could be associated with postpartum depressive/physical symptoms were not included in this study or adjusted in the regression models. For example, stressful life events, daily routine (e.g., sleeping), sociocultural factors (e.g., adherence to doing-the-month practice), and parenting could have resulted in residual confounding.

## Conclusions

Domestic decision-making power and social support are two factors that protect against postpartum depressive and physical symptoms among pregnant women. To decrease postpartum depressive and physical symptoms, policymakers and health professionals should focus on improving women’s participation in decision-making in the household and provide interventions related to social support and empowerment for perinatal women. Future studies could design interventions to confirm the causal relationship. The effect of domestic decision-making power on postpartum depressive and physical symptoms was less salient among immigrant women; nonetheless, immigrant women had lower domestic decision-making power. The lack of significant association between domestic decision-making power and postpartum physical/depressive symptoms among immigrants could be related to healthy migrant effect and/or lower expectation toward domestic decision-making power among immigrants. Empowerment issues among immigrants still need to be addressed. Culturally tailored programs and supportive strategies could be developed for immigrant women.

## Supporting information

S1 File (SAV)
